# Free access to antiretroviral treatment and protection against the risk of catastrophic health expenditure in people living with HIV: evidence from Cameroon

**DOI:** 10.1186/s12913-021-06331-5

**Published:** 2021-04-07

**Authors:** Marwân-al-Qays Bousmah, Marie Libérée Nishimwe, Christopher Kuaban, Sylvie Boyer

**Affiliations:** 1 Aix Marseille University, INSERM, IRD, SESSTIM, Sciences Economiques & Sociales de la Santé & Traitement de l’Information Médicale, Marseille, France; 2 Centre Population et Développement (Ceped), Institut de recherche pour le développement (IRD) & Université de Paris, Inserm ERL 1244, 45 rue des Saints-Pères, 75006 Paris, France; 3 Faculty of Medicine and Biomedical Sciences, University of Yaoundé, Yaoundé, Cameroon

**Keywords:** HIV, Catastrophic health expenditure, Costs, Treatment, Free antiretroviral treatment, Policy evaluation, Poverty, Cameroon

## Abstract

**Background:**

To foster access to care and reduce the burden of health expenditures on people living with HIV (PLHIV), several sub-Saharan African countries, including Cameroon, have adopted a policy of removing HIV-related fees, especially for antiretroviral treatment (ART). We investigate the impact of Cameroon’s free antiretroviral treatment (ART) policy, enacted in May 2007, on catastrophic health expenditure (CHE) risk according to socioeconomic status, in PLHIV enrolled in the country’s treatment access program.

**Methods:**

Based on primary data from two cross-sectional surveys of PLHIV outpatients in 2006–2007 and 2014 (i.e., before and after the policy’s implementation, respectively), we used inverse propensity score weighting to reduce covariate imbalances between participants in both surveys, combined with probit regressions of CHE incidence. The analysis included participants treated with ART in one of the 11 HIV services common to both surveys (*n* = 1275).

**Results:**

The free ART policy was associated with a significantly lower risk of CHE only in the poorest PLHIV while no significant effect was found in lower-middle or upper socioeconomic status PLHIV. Unexpectedly, the risk of CHE was higher in those with middle socioeconomic status after the policy’s implementation.

**Conclusions:**

Our findings suggest that Cameroon’s free ART policy is pro-poor. As it only benefitted PLHIV with the lowest socioeconomic status, increased comprehensive HIV care coverage is needed to substantially reduce the risk of CHE and the associated risk of impoverishment for all PLHIV.

**Supplementary Information:**

The online version contains supplementary material available at 10.1186/s12913-021-06331-5.

## Introduction

Universal health coverage is a major target for health reform in many countries and a priority of the Sustainable Development Goals. The development of health insurance schemes and the removal of user fees for specific populations and/or key health services are considered core actions to achieve this target [1].

People living with HIV (PLHIV) constitute an especially vulnerable population as they bear particularly large disease-related health expenses which may severely affect their household welfare [2]. Catastrophic health expenditure (CHE) - which assess the financial hardship caused by out-of-pocket payments for health on household welfare - is very high in this population, although some studies suggest that access to antiretroviral treatment (ART) and longer time on ART may decrease its risk [3, 4]. To foster access to care and reduce the burden of health expenditures on PLHIV, several sub-Saharan African countries, including Cameroon, have adopted a policy of removing ART-related fees. However, evidence for this policy’s success is limited. In particular, little is known about the effects of free ART on CHE according to PLHIV socioeconomic status, and especially on the financial risks faced by the poorest groups in this vulnerable population. This information is crucial for decision makers, to identify the strengths and weaknesses of current policy and tailor future policy accordingly.

The Cameroonian health system is mainly financed by private health expenditures through out-of-pocket payments, the latter accounting for 71% of current health expenditures in 2017 [5]. Following a recent multi-country study which estimated CHE incidence in 2014 in Cameroon’s general population at 3% - using the common thresholds of 10% of total consumption or 40% of non-food consumption - the country was classified in the second highest quintile of CHE incidence worldwide [6].

Furthermore, Cameroon is one of the countries most affected by the HIV epidemic in West and Central Africa, with a prevalence estimated at 3.6% in 2018 in adults aged 15–49 years [7]. In 2001, the government launched a national treatment access program based on a strategy of HIV care decentralization, whereby ART was delivered at the district level [8]. This provided healthcare facilities a relatively large degree of autonomy in terms of the design of user fee schemes, resulting in price differentiation in healthcare facilities across the country. However, ART prices were fixed and were subsidized by the government [9]. A study conducted in 2006–2007 showed that, overall, decentralization had a protective effect on the risk of CHE in PLHIV [9]. However, 39.6% of this population risked CHE at the threshold of 20% of household income, with an even higher risk for the poorest individuals. In May 2007, the national authorities decided to provide ART for free to facilitate access to treatment and remove financial barriers. However, user fees continued to apply to all other HIV services, including consultations and biological tests.

This study aimed to explore the differential effects of the free ART policy on the risk of CHE according to socioeconomic status in PLHIV enrolled in Cameroon’s ART access program.

## Data

### Study design

We used data from two cross-sectional surveys conducted in 2006–2007 and 2014 in PLHIV outpatients attending HIV services participating in the Cameroonian ART access program. The first survey, entitled EVAL (ANRS-12116), included 3151 HIV-positive outpatients consulting in 27 HIV services in 6 of the country’s 10 regions [8]. The second survey, entitled EVOLCam (ANRS-12288), included 2130 participants consulting in 19 HIV services in the Centre and Littoral regions. Eleven of the latter also participated in the 2006–2007 survey [10]. In order to study evolutions in the program over time, both surveys used the same design and procedures, which are described in detail elsewhere [11].

Briefly, outpatients diagnosed HIV-positive at least 3 months previously and aged ≥21 years were randomly selected for inclusion. All participants provided written consent before data collection. They answered a face-to-face questionnaire documenting socioeconomic characteristics, household consumption, healthcare use and related expenditures. Blood samples were also collected and analyzed in a reference laboratory in Yaoundé for viral load and CD4 cell count measurements. Clinical data were obtained from both clinical examination and retrospective medical files using a standardized medical questionnaire.

The EVAL (ANRS-12116) and the EVOLCam (ANRS-12288) surveys were both approved by the Ministry of Public Health in Cameroon and the Cameroonian National Ethics Committee.

### Study population

The study population for the present analysis included participants treated with ART for at least 1 month either in 2006–2007 (EVAL survey) or in 2014 (EVOLCam survey) in one of the 11 HIV services participating in both surveys (*n* = 1275, i.e., 615 and 660 participants, respectively).

### Variables

The outcome of interest was a binary variable measuring CHE incidence. Using a standard method, CHE was defined as out-of-pocket payments for health accounting for 40% or more of a household’s capacity to pay (i.e., the income remaining after subsistence needs are met) [12].

The following socioeconomic and clinical variables were included in the analysis as determinants of CHE: wealth level (which depending on the model was assessed either by i) the log of the monthly equivalized consumption expenditure (continuous variable) or ii) the monthly equivalized consumption expenditure in deciles (categorical variable)), age, gender, level of formal education, marital status, being the head of the household, having an economic activity, time (in months) since ART initiation and CD4 cell count at the time of the survey (< 200 versus ≥ 200cells/mm^3^). We also included i) a decentralization variable to indicate each HIV service’s decentralization level (i.e., central or district) and ii) the survey period (2006–2007, i.e., before the free ART policy’s implementation versus 2014, i.e., after its implementation). A full description of the variables is presented in Supplementary Table S1.

## Methods

We implemented an inverse probability of treatment weighting (IPTW) technique in order to reduce covariate imbalances between ‘treated’ and ‘untreated’ individuals (i.e., participants in the 2014 survey who benefited from the free ART policy and participants in the 2006–2007 survey who did not, respectively). This methodology, which is part of the general framework developed by Rosenbaum & Rubin [13] for estimating average treatment effects (ATE), allowed us to reduce any possible bias when estimating the policy’s causal effect on CHE. IPTW is widely used for estimating ATE in the presence of selection bias related to sample selection or stratification based on covariates [14]. More specifically, it consists in weighting the ‘treated’ group by the inverse of the propensity score. The rationale behind using the inverse propensity score lies in the so-called “double robustness” result, implying that the ATE is consistent if at least one of the conditional mean functions of the response or the propensity score model is correctly specified [15].

First, we estimated the propensity score of the treatment (i.e. benefitting from the policy) using a probit model including the explanatory variables listed in the Variables subsection above. Balance properties were analyzed using a standard approach [16]. We then estimated a probit model weighted by the inverse probability of treatment to identify the determinants of CHE and more specifically the effect of the free ART policy. The latter analysis was restricted to the region of common support. Finally, two alternative model specifications were considered to investigate the impact of the policy on the risk of CHE according to socioeconomic status. The first specification included the continuous equivalized consumption expenditure variable and an interaction of this variable with the free ART policy variable, to test whether the policy had pro-poor effects (i.e., decreased the probability of CHE more in poorer individuals than in others). The second specification included the equivalized consumption expenditure variable in deciles and an interaction of this variable with the free ART policy variable, to test whether the effect of the policy differed according to socioeconomic status.

As the data source consisted of two cross-sectional surveys, one before and one after the policy’s implementation in 2007, an ‘omitted variable’ bias due to potentially unobserved factors cannot be ruled out. To limit this bias, we adjusted the different models for the level of HIV service decentralization, as a previous study conducted in the first phase of Cameroon’s ART access program showed that linkage to care at the district level was associated with a lower risk of CHE [9]. Furthermore, as the policy may have influenced CHE in ways other than direct cost reduction - for instance, by improving access to care which in turn resulted in fewer adverse health events - we also controlled for health-related variables (e.g., CD4 cell count at the time of the survey).

## Results

### Main characteristics of the study population according to each survey period

CHE incidence was 22% in 2006–2007 but only 15% in 2014 (see Supplementary Table S2). The mean monthly equivalized consumption expenditure was very similar in both periods (US$91 in 2006–2007 and US$90 in 2014). Mean age was 39 and 42 years in 2006–2007 and 2014, respectively. Finally, women accounted for 68 and 71% of the study population, respectively.

### Inverse probability of treatment weighting ATE estimation

Ninety-eight participants of the total 1275 participants were outside the region of common support and thus were excluded from the current analysis. The balancing property of the propensity score was satisfied (see Supplementary Table S3). Details on the inverse probability weighting estimation of the ATE are provided in Supplementary Table S4 and balancing results in Supplementary Table S5. Results from the overidentification test for covariate balance (*p* = 0.41) showed that most of the bias between ‘treated’ and ‘untreated’ individuals was removed. Our methodological choice was justified, as ATE estimation without addressing covariate imbalances between ‘treated’ and untreated” individuals yielded a significant but biased ATE of − 0.0509 (*p* = 0.047). Using the IPTW estimator, the ATE of the free ART policy on CHE was not significant.

### Effect of the free ART policy according to socioeconomic groups

**Fig. 1:**
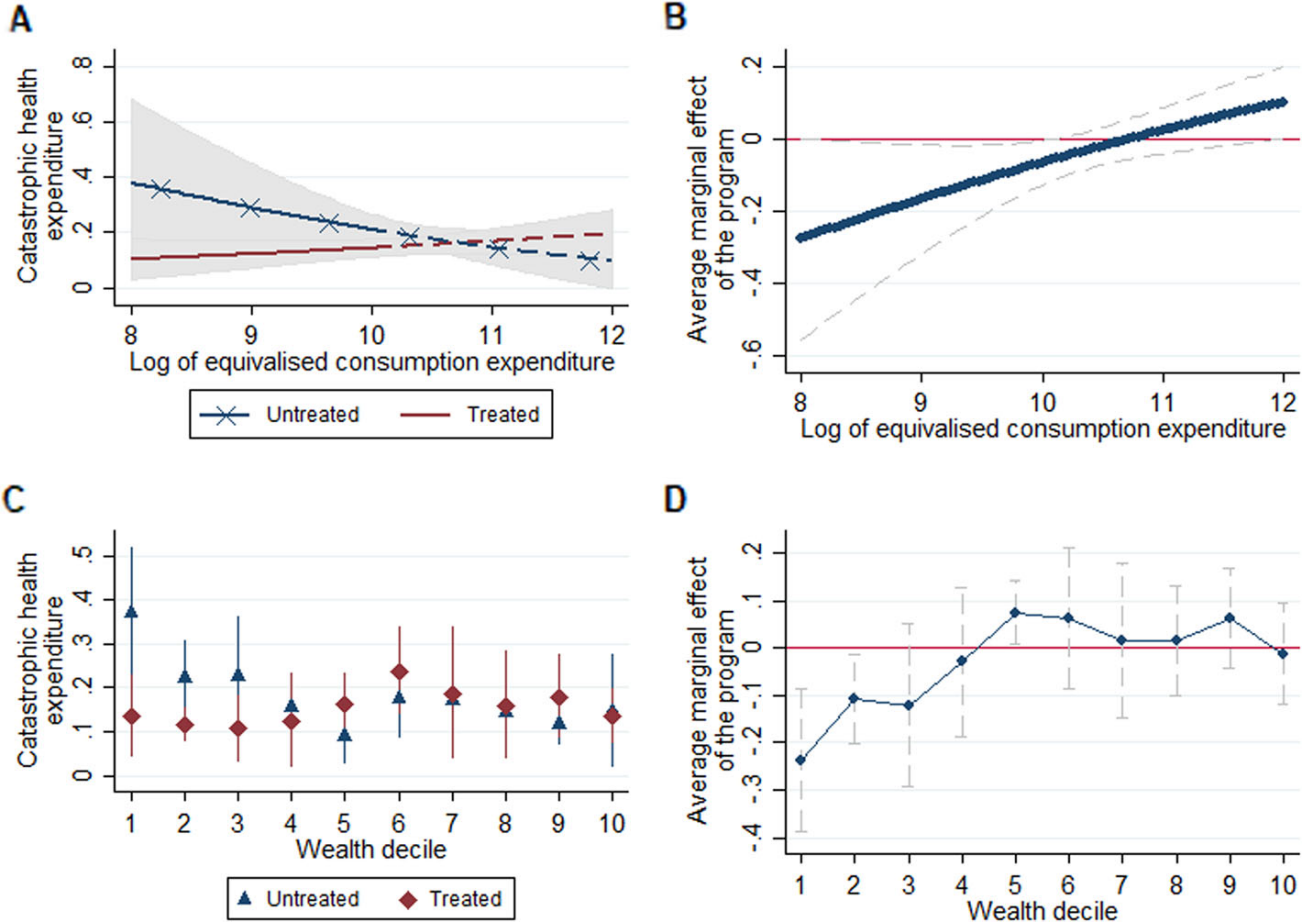
Predicted probabilities of catastrophic health expenditure and average marginal effects of the free ARV policy across wealth levels

Results are displayed in Fig. 1 and full regression results are provided in Supplementary Table S6. Panel 1.A shows the predicted CHE probabilities for the ‘untreated’ and ‘treated’ groups obtained in the first regression, with solid lines representing significant group differences (*p* < 0.05). Average marginal effects of the policy across the wealth distribution are displayed in Panel 1.B (negative effects for a specific consumption expenditure indicate a lower probability of CHE at that level). Figure 1 also shows that the policy had a significant negative average marginal effect on CHE for relatively low levels of wealth, specifically for values of the log monthly equivalized consumption expenditure between US$6/month and US$50/month. These amounts were below Cameroon’s official minimum wage in 2014 (US$62/month) [17].

The results obtained in the second regression are illustrated in Panels 1.C and 1.D which show, for each wealth decile, the predicted probabilities of CHE for both the ‘untreated’ and ‘treated’ groups and the average marginal effects of the free ART policy, respectively.

Detailed results, including predicted probabilities, average marginal effects, and tests of the equality of marginal effects across wealth deciles, are summarized in Table 1. Results obtained in the first regression were confirmed. More specifically, the free ART policy significantly reduced the probability of CHE by 23.7 percentage points (from 37.2 to 13.5%) and 10.8 percentage points (from 22.3 to 11.5%) in individuals in the lowest and second lowest wealth deciles, respectively. In addition, results showed a significant positive average marginal effect of the policy - a 7.4 percentage point increase in CHE probability - in individuals in the fifth wealth decile. No significant effect was found for the other wealth deciles.

**Table 1:** Probability of facing catastrophic health expenditure: average marginal effects of the free ARV program across wealth deciles

	Untreated	Treated	Average marginal effects of the program	Tests for the equality of marginal effects
1st decile	0.372	0.135	−0.237^*^	4th–10th
2nd decile	0.223	0.115	−0.108^*^	5th–6th, 9th
3rd decile	0.227	0.106	−0.121	5th–6th
4th decile	0.155	0.125	−0.030	1st
5th decile	0.090	0.164	0.074^*^	1st-3rd
6th decile	0.177	0.239	0.062	1st-3rd
7th decile	0.171	0.187	0.016	1st
8th decile	0.145	0.161	0.016	1st
9th decile	0.118	0.179	0.061	1st-2nd
10th decile	0.147	0.135	−0.012	1st

The tests for the equality of marginal effects report which marginal effects are significantly different (*p* < 0.05, two-tailed tests) across wealth deciles

^*^p < 0.05, two-tailed tests

## Discussion

Our findings suggest that the free ART policy introduced in Cameroon in 2007 had a different impact on the risk of CHE depending on PLHIV socioeconomic status.

More precisely, PLHIV in the lowest and second-lowest wealth deciles benefited from a 23.7 and 10.8 percentage point decrease in CHE risk, respectively. Accordingly, the policy was pro-poor. Conversely, PLHIV in the fifth wealth decile had a 7.4 percentage point increased CHE risk, while for all other wealth deciles the risk of CHE remained relatively stable before and after the introduction of the policy. These findings might be explained by the fact that concurrently with the policy’s adoption, out-of-pocket spending for certain health goods and services increased. Our data showed that expenditures for biological tests almost doubled between 2006 and 2007 and 2014 (*p* < 0.001), while other spending categories (consultation fees, hospital bills, other medications, and traditional medicine) remained stable (see Supplementary Table S2). However, this increase (whether demand- or supply-driven) was mostly observed in the middle socioeconomic group. The significant increase in CHE risk observed in PLHIV in the fifth wealth decile may also be related to a phenomenon revealed by Wagstaff & Lindelow [18] in China. In that study, the authors highlighted that health insurance substantially increased the risk of high and catastrophic health spending, as individuals received more sophisticated and expensive medical care once insured.

Our findings have important implications for HIV healthcare policy. They suggest that removing ART fees is a pro-poor measure. CHE incidence significantly reduced in poorer PLHIV in Cameroon between the two study time points. Although much higher in this population in 2006–2007, it was similar or even lower to that observed in wealthier PLHIV in 2014. However, the free ART policy alone seemed to be insufficient to effectively protect all PLHIV against the financial risk related to their infection, as suggested by the continued high level of CHE incidence in 2014 across all socioeconomic groups (from 11.5% for the second lowest decile to 23.9% for the 6th decile). Increased comprehensive HIV care coverage is necessary to significantly lower the risk of CHE and related impoverishment in the PLHIV population in Cameroon and elsewhere. In 2019, the country’s health authorities took an important step in this direction by providing free comprehensive care, including consultations, biological tests and prophylaxis drugs, to all PLHIV [19]. Further research is needed to inform policymakers about the impact of the roll-out of the free HIV care policy on socioeconomic inequalities.

The removal of user fees for HIV care, initiated in Cameroon in 2007 with the adoption of free ART and pursued in 2019 with the removal of user fees for all other HIV services, raised the question of the funding of these measures and, in turn, of the budget reforms required to compensate providers for lost fee revenue [20]. Not compensating for this loss of revenue may impede the quality of care and lead to adverse effects such as drug shortages [21], which may result in increasing access to private and costly care and, in turn, increase out-of-pocket payments for health care. However, some studies suggested that the increase of public health expenditures required to fund free care may be not financially sustainable for national governments in the long term [22]. This issue is part of a more general discussion on the financing of UHC policies in developing countries. A recent study suggested that a financing of UHC through consumption taxation was the best policy option, in terms of both fiscal sustainability and intergenerational equity [23].

Our main study limitation is the risk of ‘omitted variable’ bias in the estimation of the free ART policy effect. Although we used the IPTW method to address issues related to sample selection bias and stratification, and adjusted the models for other factors that may have influenced CHE, we cannot fully exclude it. We therefore acknowledge that the estimated differences presented here may not be entirely attributable to the free ART policy.

In conclusion, Cameroon’s free ART policy seems to have significantly reduced CHE incidence for the poorest PLHIV after its introduction in 2007. The constant, and in some cases, increased spending for certain healthcare costs items such as biological tests, might explain why other socioeconomic groups did not benefit from this policy and remained at a high risk of CHE. Increased comprehensive coverage of HIV care is needed to substantially reduce the risk of CHE and associated impoverishment.

## Supplementary Information

**Additional file 1: Table S1**. Description of variables. **Table S2**. Descriptive statistics according to survey year. **Table S3**. Propensity score model: inferior bounds and number of untreated and treated individuals in each block. **Table S4**. Average treatment effect of the free ART program on catastrophic health expenditure. **Table S5**. Inverse probability of treatment weighting: covariate balance. **Table S6**. Results of the inverse probability of treatment weighting probit model for catastrophic health expenditure.

## Data Availability

The manuscript has data included as electronic supplementary material. More complete data is available from the authors upon request (contact: Marwân-al-Qays Bousmah. CEPED (UMR 196), Université de Paris, Campus Saint-Germain, 45 Rue des Saints-Pères, 75006 Paris, France. Tel.: + 33643521166. E-mail: marwan-al-qays.bousmah@ird.fr).
