# Supplementary material for: Free access to antiretroviral treatment and protection against the risk of catastrophic health expenditure in people living with HIV: evidence from Cameroon
Source: BMC Health Serv Res. 2021 Apr 7;21:313. doi: 10.1186/s12913-021-06331-5 (PMC8028721; doi:10.1186/s12913-021-06331-5)
Supplement: Supplementary file 1 — Additional file 1: Table S1. Description of variables. Table S2. Descriptive statistics according to survey year. Table S3. Propensity score model: inferior bounds and number of untreated and treated individuals in each block. Table S4. Average treatment effect of the free ART program on catastrophic health expenditure. Table S5. Inverse probability of treatment weighting: covariate balance. Table S6. Results of the inverse probability of treatment weighting probit model for catastrophic health expenditure. [file 12913_2021_6331_MOESM1_ESM.docx]

**Supplementary Material**

This appendix provides readers with additional information about the authors’ work.

Supplement to: "**Free access to antiretroviral treatment and protection against the risk of catastrophic health expenditure in people living with HIV: evidence from Cameroon**"

Authors: Marwân-al-Qays BOUSMAH, PhD^1,2^, Marie Libérée NISHIMWE, MSc^1^, Christopher KUABAN, MD^3^, Sylvie BOYER, PhD^1^

^1^ Aix Marseille University, INSERM, IRD, SESSTIM, Sciences Economiques & Sociales de la Santé & Traitement de l’Information Médicale, Marseille, France.

^2^ Centre Population et Développement (Ceped), Institut de recherche pour le développement (IRD) et Université de Paris, Inserm ERL 1244, 45 rue des Saints-Pères, 75006 Paris, France.

^3^ Faculty of Medicine and Biomedical Sciences, University of Yaoundé, Yaoundé, Cameroon.

Corresponding author: Marwân-al-Qays Bousmah. CEPED (UMR 196), Université de Paris, Campus Saint-Germain, 45 Rue des Saints-Pères, 75006 Paris, France. Tel.: +33643521166. E-mail: [marwan-al-qays.bousmah@ird.fr](mailto:marwan-al-qays.bousmah@ird.fr).

**Contents:**

Page 2: Table S1. Description of variables

Page 3: Table S2. Descriptive statistics according to survey year

Page 4: Table S3. Propensity score model: inferior bounds and number of ‘untreated’ and ‘treated’ individuals in each block; Table S4. Average treatment effect (ATE) of the program on catastrophic health expenditure (CHE); Table S5. Inverse probability of treatment weighting: covariate balance

Page 5: Table S6. Results of the inverse probability of treatment weighting probit model for catastrophic health expenditure

Page 6: References

**Table S1. Description of variables**

| **Variable** | **Type** | **Definition** |
| --- | --- | --- |
| Health expenditure, including:   - Consultation fees - Biological tests - Hospital bills - Antiretroviral drugs - Oher medications - Traditional medicine - Transportation | Continuous | Total household health expenditure over the previous four weeks |
| Out-of-pocket health expenditure | Continuous | Household’s payments at the point of service use over the previous four weeks, net of any insurance reimbursement and health-related transportation |
| Food expenditure | Continuous | Total household expenditure on food over the previous four weeks |
| Consumption expenditure | Continuous | Total household consumption expenditure on all goods and services over the previous four weeks |
| Food expenditure share | Continuous | Household’s food expenditure divided by its total consumption expenditure |
| Household size | Continuous | Total number of people living in the household (children and adults) |
| Household equivalence scale | Continuous | Household size to the power 0.56 (parameter estimated from previous studies based on household survey data) |
| Equivalized food expenditure | Continuous | Household’s food expenditure divided by the household equivalence scale |
| Equivalized consumption expenditure | Continuous | Household’s total consumption expenditure divided by the household equivalence scale |
| Wealth decile | Categorical | Using the equivali<ed consumption expenditure distribution |
| Subsistence expenditure | Continuous | Weighted average food expenditure of households whose food expenditure shares was in the 45th to 55th percentile range across the whole sample (ie, the poverty line) multiplied by the household equivalence scale |
| Capacity to pay | Continuous | Total household consumption expenditure minus subsistence expenditure, *or* minus food expenditure if food expenditure is lower than subsistence spending |
| Catastrophic health expenditure | Binary | 1 if household's out-of-pocket health expenditure exceeded 40% of its capacity to pay; 0 otherwise |
| Survey year | Binary | 2006-2007 (=reference category); 2014 |
| Age | Continuous | In years |
| Gender | Binary | Male (=reference category); Female |
| Level of formal education | Binary | 1 if the individual had high-school diploma; 0 otherwise (=reference category) |
| Head of household | Binary | 1 if individual was head of the household; 0 otherwise (=reference category) |
| Marital status | Binary | 1 if individual was married or in a common-law relationship; 0 otherwise (=reference category) |
| Economic activity | Binary | 1 if individual had an economic activity; 0 otherwise (=reference category) |
| Months since antiretroviral therapy initiation | Continuous | Number of months since initiating HIV antiretroviral therapy |
| CD4 cell count | Binary | 1 if individual had a CD4 cell count <200 cells/μl; 0 otherwise (=reference category) |
| Hospital | Categorical | Yaoundé Central Hospital (=reference category); Yaoundé General Hospital; CNPS Hospital; Edéa Alucam Hospital; Edéa District Hospital; Mbalmayo District Hospital; Njombé Saint Jean de Malte Hospital; Nylon District Hospital; Bonassama District Hospital; Jamot Hospital; Laquintinie Hospital |
| Hospital decentralization level | Binary | 1 if individual received care in a district hospital; 0 for care in a central hospital (=reference category) |
| Note: More details about the variables used to calculate catastrophic health expenditure may be found in Xu et al. [1] and Xu [2]. | | |

**Table S2. Descriptive statistics according to survey year**

| **Variable** | **2006-2007 (n=615)** | | | | **2014 (n=660)** | | | | **Difference between 2006-2007 and 2014 (p-value)^*^** |
| --- | --- | --- | --- | --- | --- | --- | --- | --- | --- |
|  | **Mean or proportion** | **Standard error** | **Min** | **Max** | **Mean or proportion** | **Standard error** | **Min** | **Max** |  |
| Health expenditure per category | 15,710 | 1,170 | 0 | 299,600 | 15,006 | 1,146 | 0 | 338,500 | 0.6676 |
| - Consultation fees | 1,278 | 77 | 0 | 23,200 | 1,288 | 92 | 0 | 30,000 | 0.9388 |
| - Biological tests | 2,101 | 426 | 0 | 150,000 | 4,023 | 502 | 0 | 175,000 | 0.0038 |
| - Hospital bills | 1,984 | 617 | 0 | 200,000 | 2,570 | 651 | 0 | 250,000 | 0.5151 |
| - Antiretroviral drugs | 4,171 | 117 | 0 | 30,000 | 10 | 6 | 0 | 3,500 | 0.0000 |
| - Oher medications | 3,061 | 484 | 0 | 250,000 | 3,077 | 420 | 0 | 150,000 | 0.9797 |
| - Traditional medicine | 546 | 340 | 0 | 200,000 | 556 | 188 | 0 | 100,000 | 0.9778 |
| - Transportation | 2,570 | 275 | 0 | 132,000 | 3,482 | 276 | 0 | 78,000 | 0.0196 |
| Out-of-pocket health expenditure | 13,140 | 1,107 | 0 | 298,000 | 11,524 | 1,115 | 0 | 337,000 | 0.3046 |
| Food expenditure | 44,378 | 1,405 | 4,000 | 320,000 | 43,724 | 1,373 | 0 | 300,000 | 0.7395 |
| Consumption expenditure | 103,418 | 3,684 | 6,000 | 975,000 | 94,911 | 2,703 | 100 | 445,000 | 0.0604 |
| Food expenditure share | 0.49 | 0.01 | 0.04 | 0.99 | 0.48 | 0.01 | 0 | 1 | 0.3568 |
| Household size | 5.48 | 0.14 | 1 | 30 | 5.13 | 0.14 | 1 | 45 | 0.0757 |
| Household equivalence scale | 2.47 | 0.04 | 1 | 6.72 | 2.38 | 0.03 | 1 | 8.43 | 0.0684 |
| Equivalized food expenditure | 18,985 | 608 | 769 | 172,967 | 19,525 | 642 | 0 | 150,000 | 0.5428 |
| Equivalized consumption expenditure | 44,959 | 1,667 | 2,497 | 448,591 | 44,344 | 1,542 | 54 | 392,000 | 0.7865 |
| Subsistence expenditure | 48,345 | 697 | 19,552 | 131,337 | 46,614 | 646 | 19,552 | 164,815 | 0.0684 |
| Capacity to pay | 68,899 | 3,392 | 600 | 935,000 | 62,545 | 2,388 | 0 | 387,500 | 0.1218 |
| Catastrophic health expenditure | 0.22 | 0.02 | 0 | 1 | 0.15 | 0.01 | 0 | 1 | 0.0005 |
| Age | 39 | 0 | 21 | 77 | 42 | 0 | 22 | 74 | 0.0000 |
| Gender | 0.68 | 0.02 | 0 | 1 | 0.71 | 0.02 | 0 | 1 | 0.3099 |
| Level of formal education | 0.41 | 0.02 | 0 | 1 | 0.28 | 0.02 | 0 | 1 | 0.0000 |
| Head of household | 0.63 | 0.02 | 0 | 1 | 0.62 | 0.02 | 0 | 1 | 0.6258 |
| Marital status | 0.56 | 0.02 | 0 | 1 | 0.59 | 0.02 | 0 | 1 | 0.3016 |
| Economic activity | 0.70 | 0.02 | 0 | 1 | 0.66 | 0.02 | 0 | 1 | 0.1548 |
| Months since antiretroviral therapy initiation | 19.03 | 0.66 | 1.03 | 137.48 | 48.94 | 1.42 | 1 | 172 | 0.0000 |
| CD4 cell count | 0.25 | 0.02 | 0 | 1 | 0.17 | 0.01 | 0 | 1 | 0.0007 |
| Hospital decentralization level | 0.30 | 0.02 | 0 | 1 | 0.52 | 0.02 | 0 | 1 | 0.0000 |
| Note: Values are presented as means or proportions (standard error, SE). Monetary amounts are provided in CFA francs.  ^*^ Two-sample t-test and McNemar's chi-squared test for mean and proportion differences between 2006-2007 and 2014, respectively. | | | | | | | | | |

**Table S3. Propensity score model: inferior bounds and number of untreated and treated individuals in each block**

| **Block** | **Inferior bound** | **Free ART policy** | | **Total** |
| --- | --- | --- | --- | --- |
|  |  | **Untreated** | **Treated** |  |
| 1 | 0.1056645 | 31 | 11 | 42 |
| 2 | 0.1666667 | 183 | 83 | 266 |
| 3 | 0.3333333 | 177 | 82 | 259 |
| 4 | 0.5 | 100 | 129 | 229 |
| 5 | 0.6666667 | 25 | 43 | 68 |
| 6 | 0.75 | 13 | 63 | 76 |
| 7 | 0.8333333 | 7 | 230 | 237 |
| **Total** | | 536 | 641 | 1177 |
| Note: Analysis of the balancing property was restricted to “treated” and “untreated” within the region of common support. The balancing property was satisfied in each block. | | | | |

**Table S4. Average treatment effect of the free ART program on catastrophic health expenditure**

| **Estimator: Inverse probability weighting regression adjustment** | **Coefficient** | **Robust standard error** | **95% confidence intervals** | **p-value** |
| --- | --- | --- | --- | --- |
| Free ART program | -0.0341589 | 0.023198 | (-0.0796262; 0.0113084) | 0.141 |
| **Overidentification test for covariate balance**  H0: Covariates are balanced | chi2(11) = 11.391  Prob > chi2 = 0.4111 | | | |
| Note: see Imai & Ratkovic [3] for the use of the overidentification test when employing the inverse propensity score weighting method. Variables included in the regression: equivalized consumption expenditure, age, gender, level of formal education, whether the individual was the head of the household, marital status, economic activity, time since antiretroviral therapy initiation, CD4 cell count, and hospital decentralization level. Standard errors were clustered at the hospital level to account for potential within-hospital correlation. | | | | |

**Table S5. Inverse probability of treatment weighting: covariate balance**

| **Covariate** | **Standardized differences** | | **Variance ratio** | |
| --- | --- | --- | --- | --- |
|  | **Raw** | **Weighted** | **Raw** | **Weighted** |
| Equivalized consumption expenditure | 0.009884 | -0.2512604 | 1.004071 | 0.5937083 |
| Age | 0.2924249 | 0.1499795 | 1.157168 | 0.9904764 |
| Gender | 0.0290158 | 0.1078585 | 0.9754385 | 0.9317989 |
| Level of formal education | -0.2308288 | -0.1649547 | 0.8528587 | 0.924915 |
| Head of household | -0.0079861 | -0.050258 | 1.003865 | 1.028275 |
| Marital status | 0.084559 | -0.0911994 | 0.9745494 | 1.039804 |
| Economic activity | -0.0917106 | -0.0363666 | 1.075839 | 1.031192 |
| Months since antiretroviral therapy initiation | 1.054396 | -0.118932 | 4.699305 | 0.6858528 |
| CD4 cell count | -0.2026633 | 0.017262 | 0.7460813 | 1.024323 |
| Hospital decentralization level | 0.4398665 | 0.1328743 | 1.16628 | 1.072626 |

**Table S6. Results of the inverse probability of treatment weighting probit model for catastrophic health expenditure across socioeconomic groups**

|  | **Model 1** | **Model 2** |
| --- | --- | --- |
|  | **Interaction of the wealth (continuous) and program variable** | **Interaction of the wealth decile and program variable** |
|  | **Coefficient estimate** | **Coefficient estimate** |
| Free ART program | -4.043^*^  (1.660) | -0.860^***^  (0.260) |
| Log of equivalized consumption expenditure | -0.266  (0.181) |  |
| Log of equivalised consumption expenditure x Free ART program | 0.374^*^  (0.160) |  |
| Wealth decile (1^st^=reference category)  2^nd^  3^rd^  4^th^  5^th^  6^th^  7^th^  8^th^  9^th^  10^th^ |  | -0.464^*^  (0.191)  -0.451^+^  (0.250)  -0.734^***^  (0.169)  -1.082^***^  (0.248)  -0.643^**^  (0.226)  -0.666^+^  (0.364)  -0.781^**^  (0.247)  -0.914^**^  (0.282)  -0.772^+^  (0.418) |
| Wealth decile x Free ARV drug program  2^nd^  3^rd^  4^th^  5^th^  6^th^  7^th^  8^th^  9^th^  10^th^ |  | 0.364  (0.259)  0.298  (0.482)  0.686  (0.476)  1.213^***^  (0.303)  1.059^***^  (0.307)  0.893^**^  (0.324)  0.899^*^  (0.381)  1.110^***^  (0.313)  0.770^*^  (0.310) |
| Age (in years) | 0.005  (0.008) | 0.004  (0.007) |
| Female gender | 0.150^*^  (0.074) | 0.182^**^  (0.070) |
| Formal education ≥ high-school diploma | -0.232^*^  (0.104) | -0.211^*^  (0.107) |
| Head of household | 0.251^+^  (0.136) | 0.285^*^  (0.115) |
| Married or in common-law union | 0.024  (0.130) | 0.028  (0.132) |
| Economic activity | -0.249^*^  (0.109) | -0.259^*^  (0.112) |
| Time since antiretroviral therapy initiation (in months) | -0.006^***^  (0.002) | -0.007^***^  (0.002) |
| CD4 cell count < 200 cells/mml | 0.357^***^  (0.090) | 0.351^***^  (0.088) |
| District hospital decentralization level | -0.162  (0.143) | -0.169  (0.139) |
| Constant | 1.827  (1.877) | -0.340  (0.320) |
| Notes: 1176 observations in each regression. Standard errors (clustered at the hospital level to account for potential within-hospital correlation) in brackets..  x = interaction term, ^+^ p < 0.10, ^*^ p < 0.05, ^**^ p < 0.01, ^***^ p < 0.001. | | |

**References**

1. Xu K, Evans DB, Kawabata K, Zeramdini R, Klavus J, Murray CJL. Household catastrophic health expenditure: a multicountry analysis. Lancet. 2003;362:111–7.

2. Xu K. Distribution of health payments and catastrophic expenditures - Methodology. Geneva, Switzerland: World Health Organization; 2005.

3. Imai K, Ratkovic M. Covariate balancing propensity score. J R Statist Soc B. 2014;76:243–63.
